# Characterization of Cyclooxygenase-2 and its induction pathways in response to high lipid diet-induced inflammation in *Larmichthys crocea*

**DOI:** 10.1038/srep19921

**Published:** 2016-02-01

**Authors:** Tianjiao Wang, Jing Yan, Wei Xu, Qinghui Ai, Kangsen Mai

**Affiliations:** 1 The Key Laboratory of Aquaculture Nutrition and Feed (Ministry of Agriculture) and Key Laboratory of Mariculture (Ministry of Education), Ocean University of China, 5 Yushan Road, Qingdao, Shandong 266003, People’s Republic of China

## Abstract

The present study was conducted to investigate the effects of a high-lipid diet (HLD) on cyclooxygenase (Cox)-2 expression and the signalling pathways related to low-grade inflammation in the large yellow croaker (*Larmichthys crocea*). An isolated 2508 bp cDNA clone of *cox-*2 contained an open reading frame spanning 1827 bp encoding a protein with 608 amino acid residues. The over-expression of *cox-2* was consistent with the activation of c-Jun N-terminal kinases (JNKs) and p38 mitogen-activated protein kinase (MAPK) in HLD-fed fish. The activation of the activator protein-1 (AP-1) and the nuclear transcription factor kappa-B (NF-κB) signalling pathways in HLD-fed fish and the significant increase of *cox-2* promoter-luciferase activity *in vitro* indicated that AP-1 and NF-κB could combine *cox-2* promoter to promote its transcription, respectively. Together, HLD-induced inflammation up-regulates *cox-2* expression via JNKs and p38 MAPK-dependent NF-κB and AP-1 pathways. The present study provides important insight into the signal transduction pathways involved in HLD-induced inflammation, which is detrimental to the health and production of fish as well as to the health of fish consumers.

A high lipid diet (HLD), containing higher lipid concentration than appropriate (the control), has been increasingly used for the development of cost-effective aquaculture, mainly due to its protein sparing effect^1^, meaning that HLD could be more beneficial for improving protein utilization in tissue synthesis than for acting as an energy source and could maximize nitrogen retention in fish. However, an HLD often leads to abnormal lipid deposition with chronic and low-grade inflammation of farmed fish^2^, which is detrimental to the health and production of fish and the health of fish consumers^3^.

Cyclooxygenase (Cox)-2 is a prostaglandin synthesis enzyme that plays a key role in inflammation in fish^4^,^5^. Cox-2 is expressed at low levels in resting cells and can be markedly induced by lipid peroxides, as well as pro-inflammatory and mitogenic stimuli^6^,^7^,^8^,^9^. Previous reports in mammals have revealed that the increased expression of *cox-2* is strongly correlated with HLD-induced inflammation^10^. However, no information is available regarding the regulation of *cox-2* in HLD-fed fish.

In mammals, HLD-induced inflammation leads to the activation of many transcription factors such as nuclear transcription factor kappa-B (NF-κB) and transcriptional factor activator protein-1 (AP-1)^11^. The phosphorylation of IκB by cytoplasmic IκB-kinase (IKK) complexes liberates NF-κB, which subsequently migrates to the nucleus and binds to the *cis*-acting κB enhancer element of target genes^12^,^13^. The double phosphorylation of c-Jun by c-Jun N-terminal kinases (JNKs) activates c-Jun, which translocates into the nucleus and then binds Jun or Fos family members to form the AP-1 early response transcription factor^14^,^15^. In mammals, *cox-2* is one of the downstream targets of both NF-κB and AP-1^16^,^17^. However, no information is available on the signalling cascades of NF-κB and AP-1 or their regulatory effects on *cox-2* in fish.

The mitogen-activated protein kinases (MAPKs) pathways are well conserved across vertebrates, and all members of the MAPK family have been identified in fish. These members include extracellular signal-regulated kinases 1&2 (ERK1/2) that are related to cell proliferation and survival^17^,^18^, JNKs and p38 MAPK that are related to inflammation and programmed cell death^19^,^20^. Several studies in mammals have reported that MAPKs could be activated by a HLD, and transduce the HLD stimulus to the nucleus by mediating downstream signalling cascades, such as NF-κB and AP-1^21^. However, little information is available on the activation of MAPKs and their effect on downstream transcription factors in response to a HLD in fish.

Large yellow croaker (*Larmichthys crocea*), with its high level of production and critical role in human food or health^22^, has been widely cultured in southeast China. The use of a HLD has become more common in large yellow croaker commercial diets, because of its protein sparing effect. The long-term use of a HLD abnormally increases lipid accumulation in the liver^23^ and the level of inflammation characterized by increased immune-related gene expression^8^ and mitochondrial function disorder^3^. Furthermore, previous studies have indicated that lipid deposition and inflammatory signals of the large yellow croaker are strikingly similar to those of other fish species and mammals^23^. Thus, the large yellow croaker is an appropriate fish species to investigate the mechanisms of HLD-induced inflammation.

Studies on nutrition of this fish have been intensively conducted in recent years^24^, but no information is available on the molecular mechanism of HLD-induced inflammation. Thus, in this study, *cox-2* from large yellow croaker was cloned and characterized, and the effect of a HLD on *cox-2* expression and the signalling transduction pathway involved were investigated.

## Results

### Characterization of full-length *cox-2* from large yellow croaker

**Figure 1:**
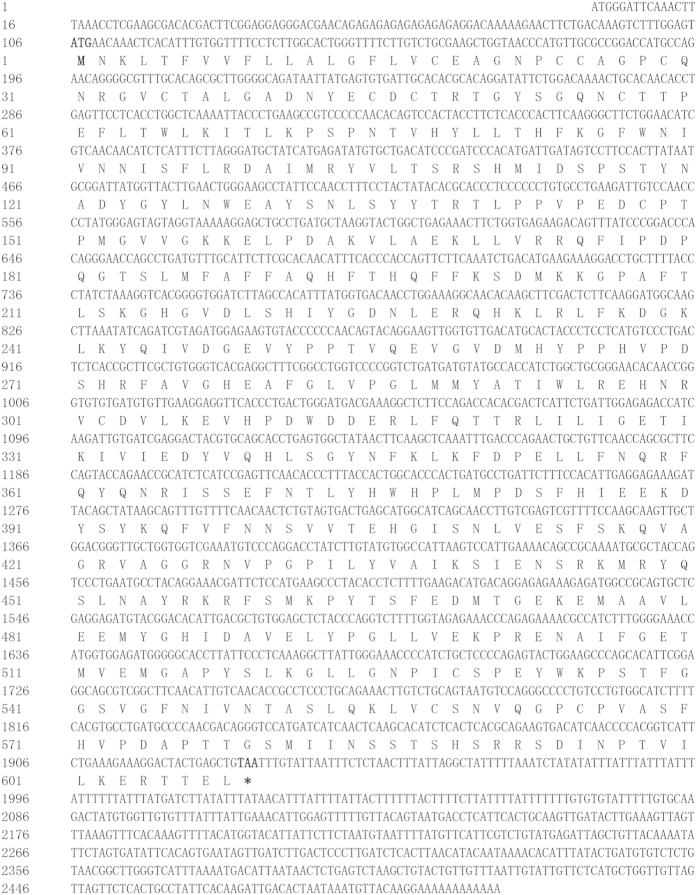
Nucleotide and deduced amino acid sequences of *cox-2*. The deduced amino acid sequences are shown below the cDNA sequences. The initiation codon (ATG) and the stop codon were characterized in bold.

The full-length cDNA of *cox-2* from large yellow croaker was 2508 bp (GenBank Accession No. KP259877), including a 5′ untranslated terminal region (UTR) of 106 bp, a 3′ UTR of 575 bp, and an open reading frame (ORF) of 1827 bp encoding a polypeptide of 608 amino acid residues with a predicted molecular weight of 69.03 KDa and a theoretical isoelectric point of 6.74 (Fig. 1).

### Multiple sequence alignment analysis and phylogenetic analysis of Cox-2

**Figure 2:**
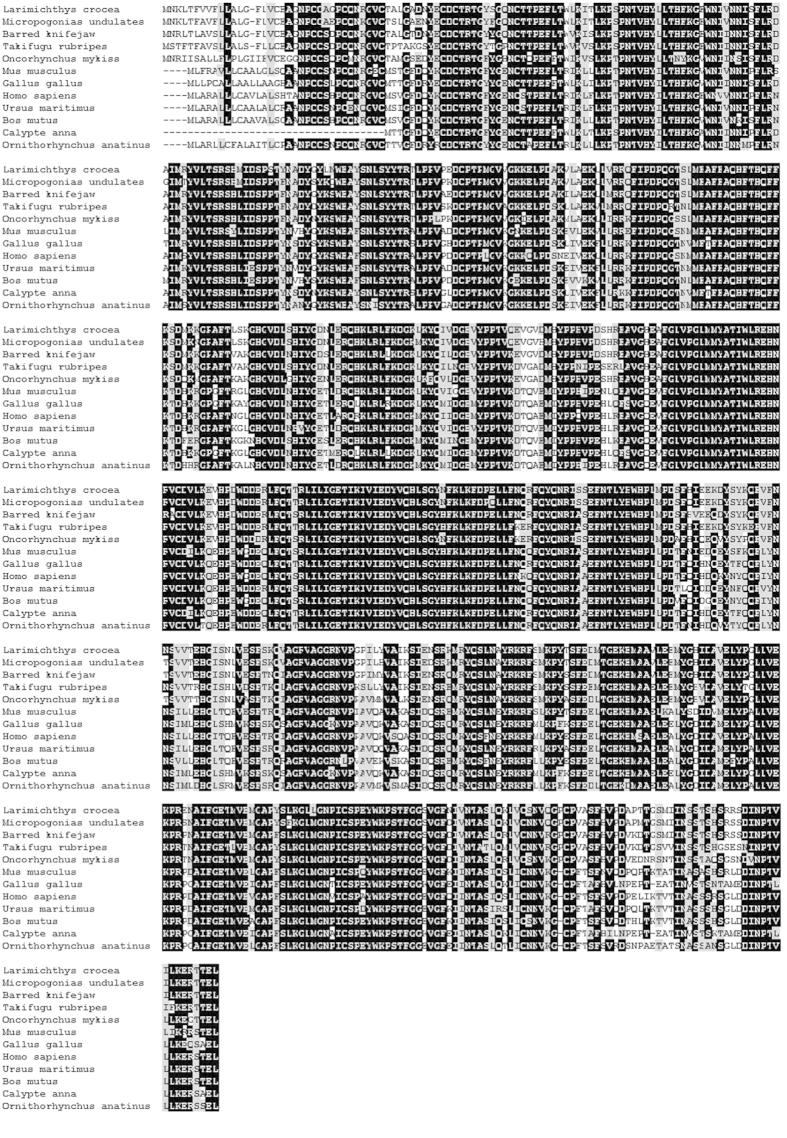
Multiple sequence alignment of Cox-2 and other invertebrate Cox-2. Alignment was performed using ClustalW2. Identical residues are indicated in black, and similar residues in light gray. Dashes indicate gaps. Identities are shown as black boxes and shaded boxes represent similar amino acids.

**Figure 3:**
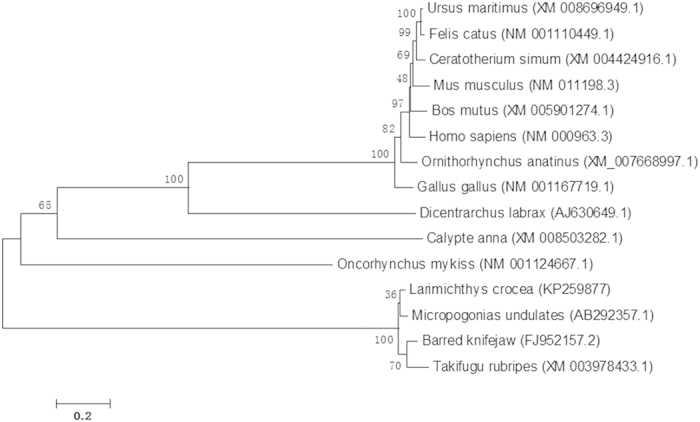
Phylogenetic tree of Cox-2. The phylogenetic tree was constructed using MEGA 4.0 software by the Neighbor-joining method based on sequence alignment using ClustalW2 and 1000 replications of bootstrap. The scale bar indicated a branch length of 0.2. Amino acid sequences of Cox-2 are obtained from invertebrate and vertebrate animals.

The BLAST analysis revealed that Cox-2 of the large yellow croaker shared high identity with the known Cox-2 of teleosts including Atlantic croaker (*Micropogonias undulatus*), 95%; rock bream (*Oplegnathus fasciatus*), 92%; red seabream (*Pagrus major*), 91%; bicolour damselfish (*Stegastes partitus*), 91%; Antarctic rock cod (*Notothenia coriiceps*), 89%; and giant killifish (*Fundulus heteroclitus*), 88%. All marine fish species and freshwater fish species clustered together and formed a sister group to the branch of mammal species such as human and mouse (Fig. 2 and 3).

### Tissue distribution of *cox-2* in the large yellow croaker

**Figure 4:**
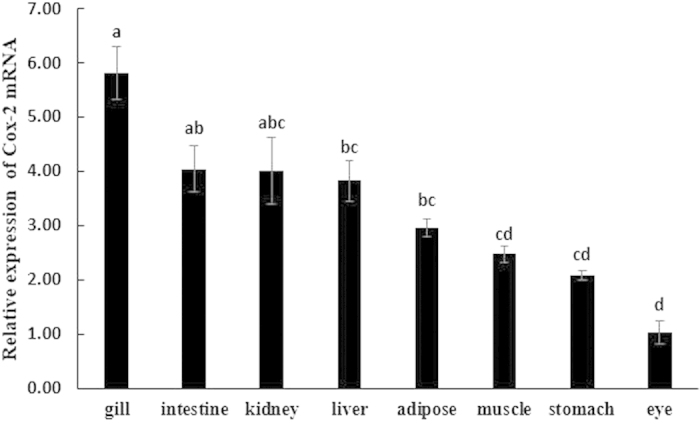
Tissue distribution of *cox-2* in large yellow croaker. Relative *cox-2* mRNA expression was determined by quantitative realtime PCR (qRT-PCR) and expressed relative to β-actin levels. Results are expressed as means ± S.E.M. (n = 3). Different letters above the bars denote significant differences among tissues at the *P* < 0.05 level (*P* = 0.000) as determined by one-way ANOVA followed by Tukey’s test (SPSS).

The tissue-specific expression of *cox-2* was examined in multi-tissues, including gill, intestine, kidney, liver, adipose, muscle, stomach and eye. The *cox-2* transcripts were broadly expressed in all of the detected tissues. The highest expression was found in gill, followed by intestine, kidney, liver, adipose, muscle and stomach, whereas the lowest expression level was observed in eye (Fig. 4).

### *Cox-2* and pro-inflammatory cytokine expression in response to dietary fatty levels

**Figure 5:**
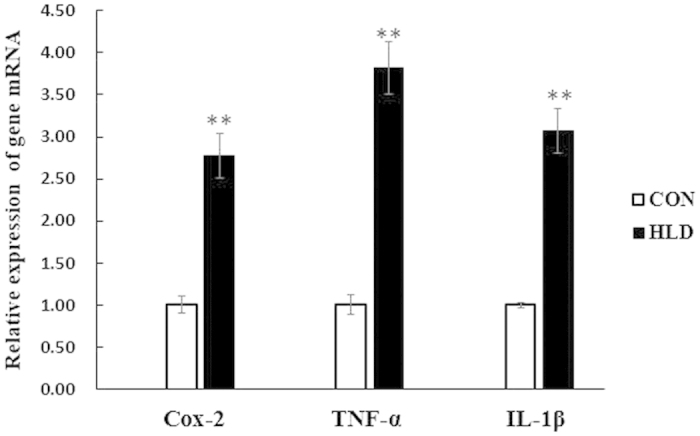
Relative *cox-2*, TNFα and IL-1β mRNA levels. Relative *cox-2*, TNFα and IL-1β mRNA levels were evaluated by quantitative realtime PCR (qRT-PCR) and expressed relative to β-actin levels in the liver of experimental fish. Results are expressed as means ± S.E.M. (n = 3). *P  < 0.05, **P  < 0.01.

**Figure 6:**
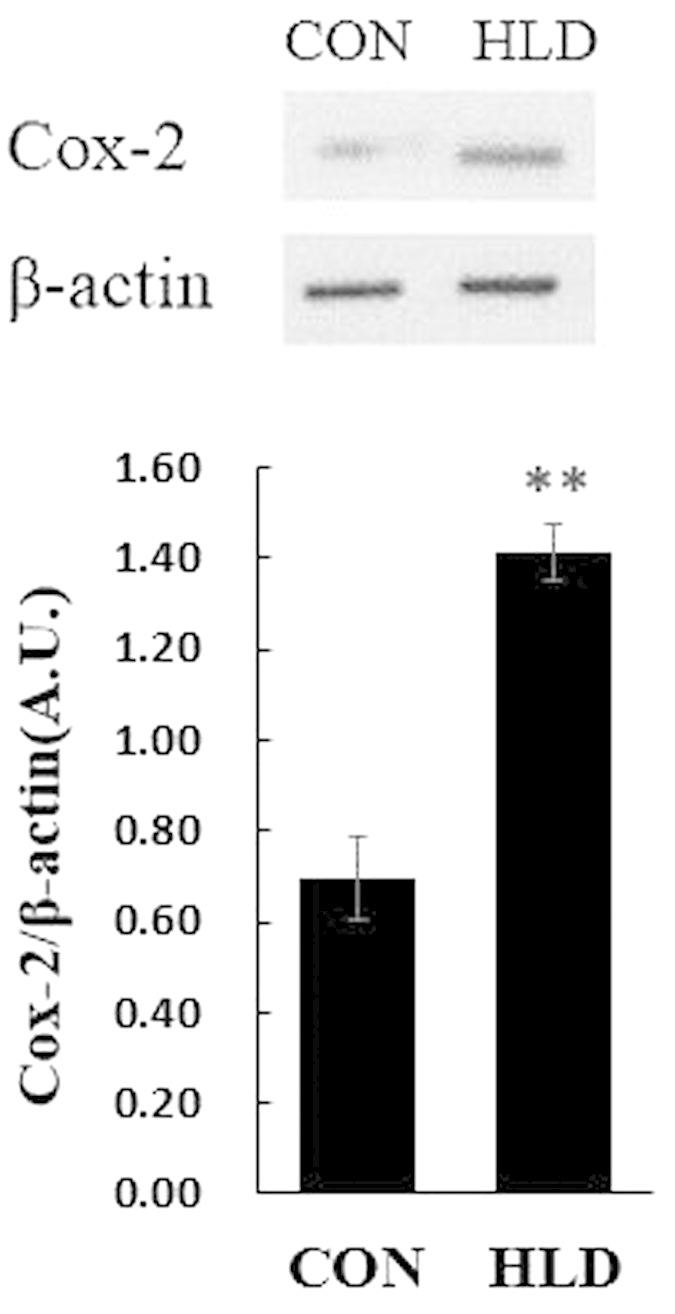
The Cox-2 protein expression levels. The Cox-2 protein expression levels were evaluated by Western blot and expressed relative to β-actin levels in the liver of experimental fish. Data are expressed as A.U. of the Western blots and are depicted as a ratio of Cox-2 to β-actin, (n = 3 in each group). All data are presented as mean ± S.E.M. *P < 0.05, **P < 0.01.

Compared with that in the control group, *cox-2* transcript levels were up-regulated in the liver of HLD fish by approximately 2.78-fold (*P* < 0.05) (Fig. 5). The levels of *cox-2* translation paralleled its transcript levels (Fig. 6). The transcript expression levels of the pro-inflammatory cytokine TNFα and IL-1β were also up-regulated in HLD fish compared with the control group (*P* < 0.05) (Fig. 5). These data indicate that *cox-2* might be induced by an HLD and may play a critical role in inflammation.

### MAPK activity, AP-1 and NF-κB signal pathways involved in dietary lipid level-induced *cox-2* expression

**Figure 7:**
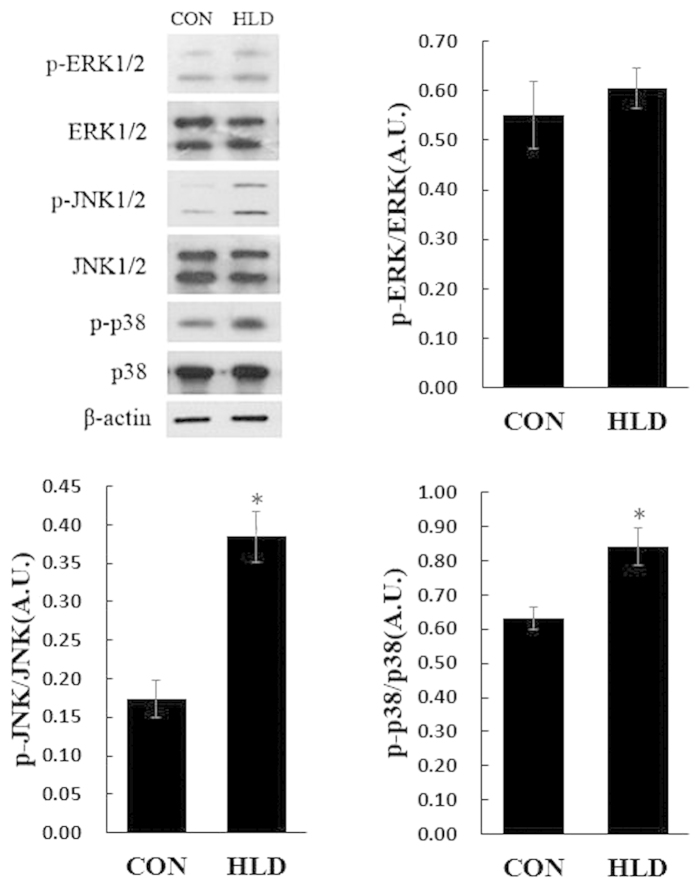
HLD activates the phosphorylation of JNK1/2 and p38 MAPK but not ERK1/2. Quantitative results of p-ERK1/2, p-JNK1/2 and p-p38 protein levels, which were adjusted with the total ERK1/2, p38, and JNK1/2 protein levels in the liver of experimental fish were analysed using Western blot. Data are expressed as A.U. of the Western blots and are depicted as a ratio of p-ERK1/2 (pThr202/Tyr204) to total ERK1/2, p-JNK1/2 (pThr183/Thr185) to total JNK1/2, and p-p38 (pThr180/Thr182) to total p38 (n = 3 in each group). All data are presented as mean ± S.E.M. *P < 0.05, **P < 0.01.

**Figure 8:**
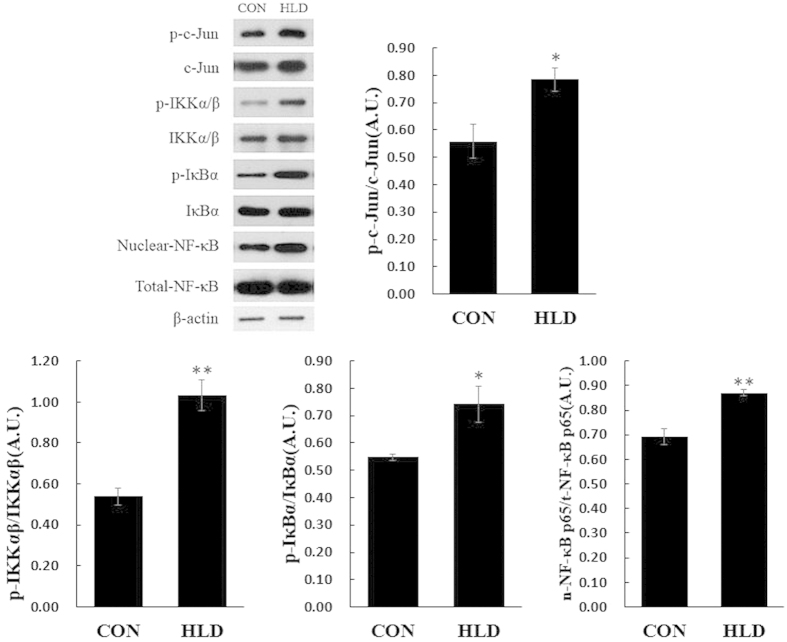
HLD activates the NF-κB and AP-1 pathway. Quantitative results of p-c-Jun, p-IKKα/β, p-IκBα protein levels, which were adjusted with the total c-Jun, IKKα/β and IκBα protein levels, were analysed using Western blot. Nuclear NF-κB p65 protein levels which were adjusted with the total NF-κB p65 protein levels, were also analysed using Western blot in the liver of experimental fish. Data are expressed as A.U. of the Western blots and are depicted as a ratio of p-c-Jun (pSer73) to total c-Jun, p-IKKα/β (pSer176/Ser180) to total IKKα/β, p-IκBα (pSer32/Ser36) to total IκBα and nuclear-NF-κB p65 to total NF-κB (n = 3 in each group). All data are presented as mean ± S.E.M. *P < 0.05, **P < 0.01.

The HLD increased the phosphorylation of JNK and p38 MAPK in HLD fish compared to the control group, whereas no significant effect on the phosphorylation status of ERK1/2 was observed among the different lipid levels (Fig. 7). No significant difference in ERK1/2, JNK and p38 total protein was observed among the different dietary lipid levels. Therefore, it is likely that JNK and p38 MAPK but not ERK 1/2 up-regulated *cox-2* expression. The phosphorylation of c-Jun was increased by the HLD, indicating the nuclear translocation level of c-Jun. Similarly, the HLD increased IKKα/β and IκBα protein phosphorylation, which induced the degradation of cytoplasmic IκBα and subsequently increased the nuclear level of NF-κB subunits p65. These data indicate that the HLD stimulated the expression of *cox-2* depending on the AP-1 and NF-κB pathways via the activation of the JNK and p38 MAPK pathways (Fig. 8).

### Dual-luciferase reporter assays

**Figure 9:**
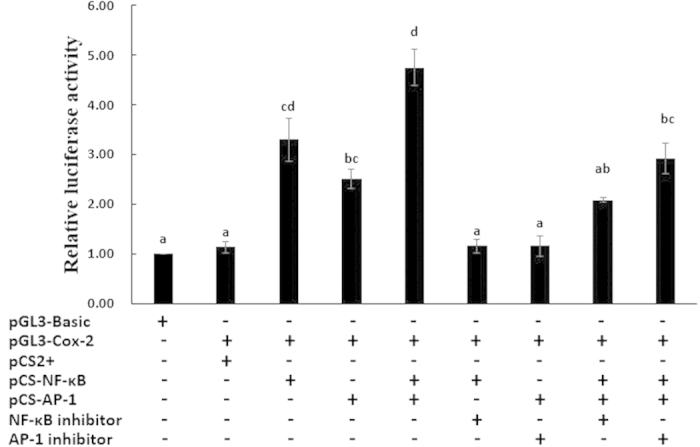
Relative dual-luciferase activity analysis of NF-κB and AP-1 in the *cox-2* promoter in HEK293 cells. The bars indicated relative luciferase activity (n = 3). PRL-CMV and pGL3-Basic used as control. The amount relative to the internal control is expressed as mean ± S.E.M. (n = 3). Significant differences across control were indicated.

Compared with the empty vector pCS2+, the expression of recombinant pCS-NF-κB and pCS-AP-1 resulted in 2.91- and 2.22-fold (*P* < 0.05) increases in pGL3-Cox-2 expression, respectively, and the combination of pCS-NF-κB and pCS-AP-1 resulted in a 4.15-fold (*P* < 0.05) increase in pGL3-Cox-2 expression. Compared to the combination of pCS-NF-κB and pCS-AP-1 without an inhibitor, the combination of pCS-NF-κB and pCS-AP-1 with a NF-κB or AP-1 inhibitor resulted in a significant decrease in pGL3-Cox-2 expression. Compared to pCS-AP-1 or pCS-NF-κB independent treatments, no significant differences were observed in pGL3-Cox-2 expression of the combination of pCS-NF-κB and pCS-AP-1 with a NF-κB or AP-1 inhibitor treatment. These results indicate that both NF-κB and AP-1 activate the expression of luciferase reporter genes, suggesting that they could promote the transcription of *cox-2* and that their regulation could be regarded as independent actions (Fig. 9).

## Discussion

The ORF of *cox-2* encodes a 608 amino acid polypeptide, which showed a high similarity to Cox-2 from other fish and mammals. The evolutionary relationship of Cox-2 is consistent with traditional taxonomy. Additionally, the highest *cox-2* expression was found in gills, a primary defence against external pathogens, thus indicating that *cox-2* may play a significant role in innate immunity.

Previous reports on fish species have indicated that *cox-2* is expressed at low levels in resting cells and is significantly induced by various fatty acids, such as saturated fatty acids^8^ and n-6 polyunsaturated fatty acids (PUFAs)^9^. Saturated fatty acids are positively associated with inflammation, mainly due to the significant effects of palmitic and stearic acids. Many studies have demonstrated that saturated fatty acids increase the saturation of membrane phospholipids^30^, initiate the unfolded protein response^31^, lead to endoplasmic reticulum stress^32^, affect mitochondrial metabolism^33^ and promote ROS accumulation^34^. In general, n-6 PUFAs have pro-inflammatory activity and can play important roles in immune function. Human inflammatory cells typically contain high proportions of the n-6 PUFA arachidonic acid (20:4n-6) and low proportions of n-3 PUFA, mainly because arachidonic acid is the precursor of two-series prostaglandins and four-series leukotrienes, which are highly-active mediators of inflammation^35^. Furthermore, some negative effects of obesity are associated with excessive levels of n-6 PUFAs^36^,^37^,^38^,^39^. In the present study, HLD-induced inflammation may be due to the high levels of saturated fatty acid and n-6 PUFAs in the HLD. Although many studies involving fatty acids induced inflammation have been reported in mammals, limited information is available in fish.

The over-expression of *cox-2* plays a crucial role in the development and progression of inflammation^40^,^41^. In the present study, high levels of *cox-2* expression were found in the liver of HLD-fed fish, and the increased *cox-2* expression was associated with increased pro-inflammatory cytokine TNFα and IL-1β transcripts (Fig. 5). In mammals, the up-regulated *cox-2* expression in liver is associated with pathological conditions, such as acute liver failure, hepatic fibrosis and cirrhosis, and hepatocarcinogenesis^41^,^42^. Given the conservation of *cox-2* function, *cox-2* over-expression in the present study supports its important role in HLD-induced hepatic inflammation.

In fish, MAPKs are associated with *cox-2* expression through several stimuli, including metals^43^,^44^ and parasites^45^, but the effects of an HLD on MAPKs remain unknown. A number of reports in mammals have demonstrated that several signal transduction pathways are simultaneously stimulated by an HLD, and these signals appear to converge at the common MAPK pathway^46^,^47^. In the present study, the HLD caused a marked increase in the level of phosphorylated JNK and p38 MAPK, whereas the phosphorylation of ERK was not affected (Fig. 7). These findings suggest that two types of MAPKs, JNK and p38 MAPK, but not ERK, are involved in the HLD-induced up-regulation of *cox-2*.

Previous studies have suggested that MAPKs activate separate effector pathways, demonstrating crosstalk between receptor systems that converged further downstream in the cell nucleus in mammals. The AP-1 and NF-κB activation can be selectively triggered by all MAPKs in a stress-dependent manner^48^,^49^. The qRT-PCR results indicated that an HLD up-regulated *cox-2* expression at the transcriptional level (Fig. 5). The activation of transcription factor AP-1 and NF-κB pathways was thoroughly studied to investigate the downstream molecular mechanism of HLD action during *cox-2* induction. In the present study, a high level of c-Jun phosphorylation was observed in HLD-fed fish, which revealed increased c-Jun nuclear translocation and AP-1 activation (Fig. 8). The HLD also activated NF-κB signalling pathways by the rapid phosphorylation of IKKα/β and the ubiquitination and proteolytic degradation of IκBα, which then resulted in the translocation of NF-κB to the nucleus (Fig. 8). In support of the findings in the present study, Dembinska *et al.* have demonstrated that the human obesity-induced inflammatory process depends on the AP-1 and NF-κB signalling pathways^50^. Therefore, the HLD-induced over-expression of *cox-2* is regulated by JNK and p38 MAPK through the AP-1 and NF-κB pathways.

The human *cox-2* promoter contains multiple potential *cis*-activating elements, including NF-κB and AP-1 binding sites^51^. The transcription factors AP-1 and NF-κB act as environmental sensors, detecting changes in the extracellular milieu through multiple signalling cascades^52^. Previous studies have demonstrated that NF-κB is not required for lipopolysaccharide (LPS)-induced *cox-2* expression in murine macrophages by the dominant negative inhibition of NF-κB and *cox-2* reporter gene activity^53^, whereas AP-1 binding activity is increased by LPS in macrophages^54^. Kim *et al.* have demonstrated that the application of a JNK inhibitor has no significant effect on papillomavirus E5-induced *cox-2* expression compared with NF-κB inhibitor and have suggested that NF-κB is a major modulator of papillomavirus E5-induced *cox-2* expression with AP-1 playing a reduced role^55^. These studies indicate that the regulation of AP-1 and NF-κB in *cox-2* transcription is not necessary through different stimuli. In the present study, dual-luciferase reporter assays showed that AP-1 and NF-κB significantly increased *cox-2* promoter-luciferase activity. As confirmed by pathway inhibitors, the regulation of *cox-2* expression by AP-1 and NF-κB occurred independently, in agreement with previous studies (Fig. 9). These findings indicate that AP-1 and NF-κB interact with cognate *cis*-acting elements within the *cox-2* promoter to promote *cox-2* transcription independently.

In conclusion, the full-length cDNA encoding Cox-2 from the large yellow croaker was cloned and characterized. The transcription factor AP-1 and NF-κB specifically bind to the *cox-*2 promoter and independently promote its expression. A HLD increases *cox-2* transcription levels, which is mediated by JNKs and p38 MAPK-dependent NF-κB and AP-1 activation. The present study provides important insight into the signal transduction pathways, which may potentially be applied to the discovery of disease mechanisms, thereby offering great benefits to the aquaculture industry and to human health.

## Materials and Method

### Ethics statement

The present study was performed in strict accordance with the Standard Operation Procedures (SOPs) of the Guide for the Use of Experimental Animals of Ocean University of China. All animal care and use procedures were approved by the Institutional Animal Care and Use Committee of Ocean University of China. Fish were anesthetized with eugenol (1:10,000) (Shanghai Reagent Corp., Shanghai, China) to minimize suffering before being assigned to cages and sampling.

### Experimental diets and feeding process

The experimental diets design and the feeding process were given in Yan *et al.*^56^. The ingredient and nutrient composition of the experimental diets are showed in Tables 1 and 2. In brief, whitefish meal and soybean meal were chosen as the main protein sources. Fish oil and soybean lecithin were chosen as the main lipid sources, which provided essential fatty acids and phosphatides, respectively. Three isoproteic (43% crude protein) diets were formulated to contain graded levels of lipid (12% and 18% on a dry basis) (Table 1). The diet with 12% crude lipid was used as the control because this dietary lipid level is optimal for the growth of large yellow croaker^57^.

At the start of the experiment, the fish were fasted for 24 h. The fish (average body weight 150.0 g) were randomly distributed into 6 cages (1.5 × 1.5 × 2.0 m) with 40 fish in each cage. Each diet was randomly allocated to triplicate cages, and the fish were hand-fed twice daily for 10 weeks.

### Sample collection

At the termination of the experiment, fish were fasted for 24 h and anesthetized with eugenol (1:10,000) (purity 99%, Shanghai Reagent, China) before sampling. Tissues including kidney, intestine, spleen, heart, liver, brain and muscle from experimental fish were collected, frozen in liquid nitrogen and then stored at −80 °C for the analysis of immune related gene expression.

### RNA extraction and cDNA synthesis

RNA extraction and cDNA synthesis has been previously described in Yan *et al.*^56^ with slight modification. In brief, total RNA was extracted from kidney, intestine, spleen, heart, stomach, liver, brain and muscle samples with Trizol Reagent (Invitrogen, USA) according to the manufacturer’s instructions, and electrophoresed on a 1.2% denaturing agarose gel to test the integrity. The quantity and quality of the total RNA were assessed using the Nano Drop^®^ ND-1000 spectrophotometer (Nano-Drop Technologies, Wilmington, DE, USA). The 260/280 nm absorbance ratios of all samples ranged from 1.90 to 2.07, indicating a satisfactory purity of the RNA samples. First-strand cDNA was reverse transcribed from the DNase-treated RNA using PrimeScript ^TM^ RT reagent Kit (Takara, Japan).

### The cloning and characterization of full-length *cox-2* cDNA

The cloning has been previously described in Dong *et al.* with slight modification^58^. Degenerate primers were designed based on highly conserved regions from the genes of other fish to amplify internal fragments, and gene-specific primers were designed based on the known sequences of the internal fragments cDNA to clone the 3′- and 5′-end by rapid amplification of cDNA ends (RACE) through a two-round PCR using the SMARTer^TM^ RACE cDNA Amplification Kit (Clontech, California, USA) (Table 3). PCR amplifications using the primers (Table 3) and Taq DNA Polymerase (Takara, alian, China) were performed with an initial denaturation at 95 °C for 3 min and 35 cycles of “95 °C for 30 s, 60 °C for 30 s, and 72 °C for 1 min”, followed by a final extension at 72 °C for 10 min. All PCR products were run on a 1.5% agarose gel, and then purified by SanPrep PCR urification Kit (Sangon Biotech, Shanghai, China). PCR products were cloned into pEASY-T1 simple cloning vector (TransGen, Beijing, China) and sequenced in BioSune (Shanghai, China).

The nucleotide and deduced amino acid sequence of *cox-2* from large yellow croaker were analyzed using BioEdit 7.0.1 and Expasysearch program (http://au.expasy.org/tools/). The sequences of *cox-2* from different species were compared by the NCBI BLAST search program. A multiple sequence alignment was performed using ClustalW (http://www.ebi.ac.uk/clustalw/) and a phylogenetic tree of Cox-2 was made by MEGA 4.0 (http://www.megasoftware.net).

### Quantitative realtime PCR (qRT-PCR) analysis

The first-strand cDNA synthesis was the same as described above. First-strand cDNA was diluted by 4 times using sterilized double-distilled water. Quantitative realtime PCR (qRT-PCR) was carried out in a quantitative thermal cycler (Mastercyclerep realplex, Eppendorf, Germany). The amplification was performed in a total volume of 25 μl, containing 12.5 μl of 2× SYBR^®^ Premix Ex TaqTMII (Takara, Japan), 9.5 μl of sterilized double-distilled water, 1 μl of each primer (10 μM) (Table 3) and 1 μl of the diluted first strand cDNA product. The real-time qPCR amplification began with 2 min at 95 °C, followed by 40 cycles of 10 s at 95 °C, 10 s at 60 °C, and 20 s at 72 °C. Melting curve (1.85 °C increment/min from 58 °C to 95 °C) was performed after the amplification phase for confirmation. Each sample was run in triplicate. Reference Beta-actin gene was used as internal control^59^. A four-fold serial dilution of the cDNA samples quantified 5 concentrations in triplicate was used to assess PCR efficiencies for each assay. The primer amplification efficiency was analyzed according to the following equation E = 10^(−1/Slope)−1^. To calculate the expression of *cox-2*, the comparative CT method (2^*−*ΔΔ*ct*^method) was used as described by Livak *et al.*^60^.

### Western blot

Total protein was extracted from the liver of experimental fish with Total Protein Extraction Kit (Applygen Technologies Inc, Beijing, China), and nuclear and cytosolic fractions were collected using a nuclear protein extraction kit (Pierce, Nashville, TN, USA), according to the instructions of the manufacturers. Protein concentration was measured using BCA kit (Pierce). Fifty micrograms of protein was loaded onto 10% sodium dodecyl sulfate–polyacrylamide gel electrophoresis (SDS-PAGE) and transferred to polyvinylidene difluoride membranes (Pall Corporation, Port Washington, NY, USA). The membranes were incubated with 5% skimmed milk for 1 h at room temperature. Immunoblots were obtained using antibodies against the following proteins: ERK1/2, ERK1/2 (pThr202/Tyr204), JNK1/2, JNK1/2(pThr183/Thr185), p38, p38 (pThr180/Thr182), IKKα/β, IKKα/β (pSer176/Ser180), IκBα, IκBα (pSer32/Ser36), c-Jun, c-Jun (pSer73), NF-κB p65 and beta-actin (Cell Signaling Technology, Danvers, MA, USA). Western blots were exposed using SuperSignal West Femto Maximum Sensitivity Substrate (Thermo Scientific, Waltham, MA, USA) and film images were scanned by Epson Perfection V33 (China).

### Plasmid constructs

For expression plasmids, NF-κB p65 ORF (GenBank Accession No. XM_010731731.1) and c-Jun ORF (GenBank Accession No. XM_010739773.1) fragments were amplified with the primers (Table 3), and subcloned into the EcoRI/Xhol site of pCS2+ vectors (Invitrogen, USA) respectively to construct pCS- NF-κB and pCS-Ap-1. For reporter plasmids, the *cox-2* promoter (a 2000bp fragment, GenBank Accession No. NW_011322590.1) was inserted into the KpnI/Xhol site of the promoterless pGL3-basic vector (Promega, USA) to construct pGL3-Cox-2. Plasmids for transfection were prepared using the EndoFree Plasmid Mini Kit (OMEGA, USA) according to the manufacturer’s instruction.

### Cell culture and transfection

HEK293T cells were maintained in Dulbecco’s Modified Eagle Medium (DMEM, Gibco, USA) supplemented with 10% foetal bovine serum (FBS, Invitrogen) at 37 °C in a humidified incubator under 5% CO_2_. For the over-expression experiments, cells were seeded in 24-well plates. Then, 24 h after seeding, 300 ng expression plasmid, 100 ng reporter gene plasmid, 10 ng pRL-CMV *Renilla* luciferase plasmid, and 1 μl Lipofectamine^TM^ 2000 were co-transfected into cells in each well in a 24-well plate. All assays were performed with three independent transfections. The BAY 11-7082 (1 μM, Sigma) and JNK-IN-8 (1 μM, Sigma) were used as NF-κB pathway and AP-1 pathway inhibitors, respectively.

### Dual-luciferase reporter assays

Firefly and renilla luciferase activities were measured using Dual-Luciferase Reporter Assay System (Promega, USA) according to the manufacturer’s instruction. Briefly, at 24 h post-transfection, HEK293 cells in 24-well plates were washed twice with 100 μl PBS, and then lysed with 2 μl 1 × passive lysis buffer at room temperature for 15 min. The cell lysate (20 μl) was transferred to a plate and 50 μl luciferase assay reagent II and 1 × Stop & Glo reagent were added in sequence. Then, firefly and *Renilla* luciferase activities were measured.

### Statistical analysis

Software SPSS 17.0 (SPSS Inc.) was used for all statistical evaluations. All data were subjected to a one-way analysis of variance (ANOVA) and followed by Tukey’s multiple-range test. The level of significance was chosen at *P* < 0.05 and the results were presented as means ± standard error of the mean.

## Additional Information

**How to cite this article**: Wang, T. *et al.* Characterization of Cyclooxygenase-2 and its induction pathways in response to high lipid diet-induced inflammation in *Larmichthys crocea. Sci. Rep.* **6**, 19921; doi: 10.1038/srep19921 (2016).

## Figures and Tables

**Table 1:** Formulation and proximate composition of the experimental diets.

	Dietary lipid levels (%)	
	CON (12)	HLD (18)
Ingredients (g/100 g)		
Fish meal^1^	39.00	39.00
Soybean meal^1^	20.00	20.00
Wheat meal^1^	23.30	23.30
Wheat starch	6.00	0.00
Fish oil^2^	6.00	12.00
Soybean lecithin^3^	1.50	1.50
Vitamin premix^4^	2.00	2.00
Mineral premix^5^	2.00	2.00
Attractant^6^	0.10	0.10
Mold inhibitor^7^	0.10	0.10
Proximate composition (g/100 g)		
Moisture	9.40	9.20
Crude protein	42.60	43.20
Crude lipid	11.50	17.80

^1^Fish meal: crude protein 74.3% dry matter, crude lipid 6.6% dry matter; soybean meal: crude protein 49.4% dry matter, crude lipid 0.9% dry matter; wheat meal: crude protein 16.4% dry matter, crude lipid 1.0% dry matter; All of these ingredients were supplied by Great Seven Biotechnology Co., Ltd., China.

^2^Fish oil: palmitic acid (16:0) content, 15.77% total fatty acids (TFA); oleic acid (18:1n-9) content, 3.29% TFA; linoleic acid (18:2n-6) content: 2.12% TFA; alpha-linolenic acid (18:3n-3) content: 0.42% TFA; ARA content, 0.23% TFA; EPA content, 13.34% TFA; DHA content, 10.53% TFA, bought from the Great Seven Biotechnology Co, Ltd, Qingdao, China

^3^Soybean lecithin: palmitic acid (16:0) content, 16. 5% TFA, stearic acid (18:0) content, 4.2% TFA, oleic acid (18:1n-9) content, 12.1% TFA, linoleic acid (18:2n-6) content: 59.2% TFA, supplied by Liuhe Feed Co., Ltd.

^4^Vitamin premix (mg or g/kg diet): cholecalciferol, 5 mg; retinol acetate, 32 mg; thiamin 25 mg;vitamin B_12_ (1%), 10 mg; riboflavin, 45 mg; pyridoxine HCl, 20 mg; ascorbic acid, 2000 mg; alpha-tocopherol (50%), 240 mg; vitamin K_3_, 10 mg; pantothenic acid, 60 mg; inositol, 800 mg; niacin acid, 200 mg; folic acid, 20 mg; biotin (2%), 60 mg; choline chloride (50%), 4000 mg;microcrystalline cellulose, 12.47 g.

^5^Mineral premix (mg or g/kg diet): CuSO_4_·5H_2_O, 10 mg; Ca (IO_3_)_2_·6H_2_O (1%), 60 mg; CoCl_2_·6H_2_O (1%), 50 mg; FeSO_4_·H_2_O, 80 mg; MgSO_4_·7H_2_O, 1200 mg; MnSO_4_·H_2_O, 45 mg; NaSeSO_3_·5H_2_O (1%), 20 mg; ZnSO_4_·H_2_O, 50 mg; CaH_2_PO_4_·H_2_O, 10 g; zeolite, 8.485 g.

^6^Attractants: glycine and betaine.

^7^Mold inhibitor: contained 50% calcium propionic acid and 50% fumaric acid.

**Table 2:** Fatty acid composition of the experimental diets (% total fatty acids).

Fatty acid	Dietary lipid level(%)	
	CON(12)	HLD(18)
C14:0	6.17	6.17
C16:0	20.43	19.52
C18:0	2.92	2.51
C20:0	1.72	2.26
∑SFA^1^	31.23	30.46
C16:1	8.83	8.68
C18:1n	19.18	22.31
C22:1n-9	1.21	1.75
∑MUFA^2^	29.22	32.74
C18:2n-6	12.53	9.86
C18:3n-6	1.43	1.46
ARA	0.71	0.65
∑n-6PUFA^3^	14.67	11.97
C18:3n-3	1.86	1.71
C18:4n-3	0.31	0.39
C20:5n-3	9.19	8.25
C22:6n-3	8.46	8.68
∑n-3PUFA^4^	2.17	2.10
n-3/n-6PUFA	0.15	0.18
∑n-3 LC-PUFA^5^	17.65	16.93
DHA/EPA^6^	0.92	1.05

^1^SFAs: saturated fatty acids.

^2^MUFAs: mono-unsaturated fatty acids.

^3^n−6 PUFAs: n−6 polyunsaturated fatty acids.

^4^n−3 PUFAs: n−3 polyunsaturated fatty acids.

^5^n−3 LC-PUFAs: n−3 long chain polyunsaturated fatty acids.

^6^DHA/EPA: 22:6n−3/20:5n−3.

**Table 3:** Sequences of the primers used in this study.

Primers	Sequence(5′-3′)
For clone	
Cox-2-F	CACCCACCAGTTCTTCAAATCTG
Cox-2-R	TGGTACAGGGTGTTGAACTCGG
Cox-2-3′F1	ACAACCTGGAAAGGCAACACAAGCT
Cox-2-3′F2	TGACTGGGATGACGAAAGGCTCT
Cox-2-5′R1	CGTGTGGTCTGGAAGAGCCTTTCGTCAT
Cox-2-5′R2	AGCCTCGTGACCCACAGCGAAGCGG
Universal Primer A Mix (UPM)	CTAATACGACTCACTATAGGGCAAGCAGTGGTATCAACGCAGAGT
Nest Universal Primer (NUP)	CTAATACGACTCACTATAGGGC
For qRT-PCR	
Cox-2-RT-F	CTGGAAAGGCAACACAAGC
Cox-2-RT-R	CGGTGAGAGTCAGGGACAT
TNFα-RT-F	ACACCTCTCAGCCACAGGAT
TNFα-RT-R	CCGTGTCCCACTCCATAGTT
IL-1β-RT-F	CAATCTGGCAAGGATCAGC
IL-1β-RT-R	GGACGGACACAAGGGTACTAA
β-actin-F	TTATGAAGGCTATGCCCTGCC
β-actin-R	TGAAGGAGTAGCCACGCTCTGT
For construction of plasmidsa	
pCS-p65-EcoRI	CGGAATTCATGGCGGATGTGT
pCS-p65-Xhol	CCGCTCGAGTCATACGGACG
pCS-c-Jun-EcoRI	CCGGAATTCATGTATACCAAGATGG
pCS-c-Jun-Xhol	CCGCTCGAGTCAGAAGGTCTGGAG
pGL3-Cox-2-KpnI	GGGGTACCTAACAAAGACAATTCACAGG
pGL3-Cox-2-Xhol	CCGCTCGAGACTCCAAAGACTTTGTC
